# What Drives Paramedics to Serve in Rural and Remote Communities?

**DOI:** 10.3390/healthcare12111062

**Published:** 2024-05-23

**Authors:** Samer Al Haliq, Talal AlShammari

**Affiliations:** Department of Emergency Medical Care, Imam Abdulrahman Bin Faisal University, Dammam 31441, Saudi Arabia

**Keywords:** paramedic motivation, rural and remote communities

## Abstract

In this study, we investigated the motivations of paramedic staff serving in rural and remote communities, given the consistent shortage of healthcare workers in these areas. Using a modified Global Motivation Scale (GMS) questionnaire, we surveyed 450 paramedics in Saudi Arabia, analyzing data from 379 respondents (response rate: 84.2%) with SPSS 29. Chi-square tests explored demographic links to motivation, and ANOVA compared mean scores across groups (*p* < 0.05). The results showed a moderate overall motivation (M = 3.37, SD = 0.82), with high intrinsic motivation (M = 3.67, SD = 0.96) and relatively high extrinsic motivation, notably in integration (M = 3.48) and identification (M = 3.41). Age and gender significantly influenced motivation (*p* < 0.05), with individuals aged 24–30 years exhibiting markedly lower motivation. ANOVA confirmed the age, gender, marital status (unmarried), and EMS experience (5–10 years) as significant factors, while the education, job title, and employment site had no significant impact. Scheffe’s post hoc test revealed age-related differences and emphasized the importance of EMS experience. This study suggests that both intrinsic factors and external pressures contribute to the lower motivation in adults in their mid-twenties in rural areas. Experience, particularly in EMS, significantly impacts motivation levels. We recommend tailored interventions that focus on intrinsic motivation and address external pressures to improve retention and care quality.

## 1. Introduction

In rural and poorly developed regions, the global health workforce consistently lacks staff [1]. Skilled healthcare personnel are essential to an effective healthcare system. A staff in the health industry that is more motivated is expected to be willing to serve in challenging situations, have lower turnover, and, in theory, offer patients better care [2]. Therefore, employers require employees who are really motivated to serve rather than those who simply show up at their places of employment [3]. An Australian study found that visiting remote communities increased students’ personal and professional motivation to serve in general practice and remote health [4]. However, one of the biggest problems facing a nation’s healthcare system is the low level of motivation among health professionals. Low motivation was ranked as the second most significant issue facing the health workforce, following staff shortages, according to a poll conducted among ministries of health across several nations [5].

Paramedics are one of the crucial components of the Emergency Medical Services (EMS) system. These staff members have undergone advanced training in patient assessment, clinical decision-making, heart rhythm interpretation, defibrillation, pharmacological treatment, and airway management. This training is completed in accordance with the national EMS instruction standards [6]. In the Kingdom of Saudi Arabia, the Saudi Red Crescent Authority (SRCA) is in charge of providing prehospital care for the general public [7]. However, its EMS personnel continue to face challenges, such as the absence of published statistics and research studies, and the difficulties facing the development of the EMS profession, despite significant progress in Saudi Arabia’s EMS programs in universities and colleges during the last decades [8,9].

The rural population of Saudi Arabia comprised 15.27% of the country’s overall population in 2022, according to the World Bank’s collection of development indicators [10]. However, there are few health professionals in rural areas, which highlights the problem of understaffing there. In addition to the lack of qualified healthcare workers, Saudi Arabia presently employs mainly non-Saudi healthcare professionals in rural areas [11]. This is a particular problem in rural regions, where there is a large non-Saudi turnover rate and the atmosphere fails to tempt Saudi health professionals due to the less attractive living conditions than those in urban areas [12]. 

Workplace motivation is essential for an organization’s growth since it enhances productivity and effectiveness [13]. The way health professionals behave at work is a reflection of their motivation, which influences how the healthcare system functions [14]. Work motivation, which is one of the variables of effectiveness as well as efficiency, also influences the sensation of career satisfaction. In the broadest concept, motivation refers to the process of starting human activities that are intended to achieve specific aims [15]. However, there is little research concerning paramedics’ motivation in Saudi Arabia’s rural and remote community settings. This research will introduce key aspects and significant elements of paramedics’ motivation that facilitate success in the paramedic profession in rural and remote community settings. To explore this topic, we aimed, in this study, to investigate what motivates paramedic staff to serve in rural and remote communities in correlation with the demographic characteristics of participants in the paramedic field in Saudi Arabia.

## 2. Materials and Methods

### 2.1. Study Design, Setting, Sample, and Sampling

To achieve the aim of the study, we employed a descriptive cross-sectional design, to investigate what motivates paramedic staff to serve in rural and remote communities in correlation with the demographic characteristics of the participants (years of paramedic experience, age, gender, marital status, education, job title, place of employment, and education). The research was carried out between October 2023 and January 2024. A snowball sample of paramedic employees in prehospital settings in the Kingdom of Saudi Arabia was used in this study. The inclusion criteria were EMS students, health assistance, EMS technicians, and specialists. The exclusion criteria were the other healthcare providers serving in EMS as administrators, physicians, pharmacists, and nurses.

In our study, we employed exponential non-discriminative snowball sampling, which began with 10 initial seeds selected based on their experience and current employment as paramedics. The initial subjects, or seeds, were recruited through direct contact with directors of paramedic employees in major hospitals and EMS units in Saudi Arabia. Criteria for these seeds included at least two years of prehospital care experience, current employment as a paramedic, and willingness to participate and refer peers. The ten seeds, selected for their extensive networks and EMS involvement, were each asked to refer at least three colleagues meeting the same criteria, continuing until the desired sample size was achieved. Detailed instructions ensured quality referrals. To control for selection bias, we employed several measures. Firstly, we ensured that the initial seeds selected for the study represented diverse geographic regions and EMS units within the Kingdom of Saudi Arabia. Additionally, we provided clear inclusion criteria to all participants, emphasizing the importance of selecting colleagues who met the same criteria. Moreover, we encouraged the participants to refer peers based on professional criteria rather than personal relationships, reducing the likelihood of bias. These measures were implemented to minimize selection bias and ensure the representativeness of our sample. We used WhatsApp to share a Google Form link, leveraging paramedics’ networks for effective and rapid dissemination. Participants were encouraged to share the link with eligible colleagues, facilitating widespread distribution.

The Steven K. Thompson sample size equation, $n=\frac{N\times p(1-p)}{\left\{N-1\times (d^{2}÷z^{2})\}+p(1-p\right)}$, was utilized to determine the sample size [16]. In this equation, *p* represents the expected population variability (0.5), *d* denotes the margin of error (0.05), *n* is the sample size, *N* represents the population size, and the *z*-score corresponds to the 95% confidence range (1.96). The population size *N* comprises Saudi Arabian licensed health assistants, emergency medical technicians, and specialists [17]. A sample size of n = 376 participants was determined to be necessary. Recruitment was conducted in educational and clinical settings of EMS practice in Saudi Arabia.

### 2.2. Instrument

This study utilized an anonymous, self-reported survey. It was adapted to be used in this study. The survey included two parts (Tables S1 and S2). The first section is a demographic data page with questions aimed at eliciting details about the demographics of participants, including age, gender, marital status, education, job title, place of employment, and years of paramedic experience. The second section includes the Global Motivation Scale (GMS) questionnaire. This self-administered questionnaire was originally developed by Guay, Vallerand, and Blanchard (2000) and was adapted for our study. It includes 18 items on a 5-point Likert-type answer scale; the higher the score, the more agreement there is, with items ranging from “Strongly disagree” (1) to “Strongly agree” (5). The instrument is intended to assess many types of long-term regulatory orientations in people: amotivation, extrinsic motivation through integrated, identified, introjected, and external regulation, and intrinsic motivation. Each subscale had sufficient internal consistency, with alpha coefficients ranging from 0.66 to 0.89 [18]. Additionally, we added one item to ask the participants’ level of agreement to recommend other colleagues serving in rural and remote communities.

To investigate the psychometric qualities of the instrument and the amount of time required to complete the questionnaire, pilot research was carried out. The overall Cronbach’s alpha value for reliability (n = 30) was 0.896. To ensure content validity, every question on the questionnaire was translated from English into Arabic and then back again by a group of highly skilled academic translators.

### 2.3. Data Collection and Analysis

This study was approved by the Institutional Review Board (IRB) of Imam Abdulrahman Bin Faisal University (IRB-2023-03-364). A digital questionnaire and cover letter were sent to the participants. In this way, before enrolling in this study, potential subjects were made completely aware of its benefits and risks. This study’s objectives were described, along with the fact that participation was optional. All subjects were also made completely aware about their rights to anonymity and confidentiality, as well as their right to voluntarily discontinue their participation whenever they wanted, without incurring any penalty for their ongoing or future labor. The subject then filled out the required surveys and was included in this study if they consented.

The data were analyzed using the Statistical Package for Social Sciences (SPSS) for Windows version 29. Data were cleaned for outliers and checked for normality and missing data. The sample characteristics and other categorical variables were described using descriptive statistics (mean, frequency, and standard deviation). The Chi-square test was used to examine the association between demographic variables (age, gender, marital status, education, job title, employment site, and years of EMS experience) and motivation to serve in rural and remote communities. Furthermore, ANOVA was used to compare mean scores across different demographic groups, to identify if there were significant differences in motivation scores based on these factors. Post hoc and multiple comparisons (Scheffe) tests were used to reveal which groups significantly differed in motivation levels. At *p* < 0.05, statistical significance was established.

## 3. Results

The survey was completed by 379 participants (response rate 84.2%). Most of the participants were younger than 24 years old, male, not married, had a bachelor’s degree, worked at the paramedic level, worked at an urban employment site, and had less than 5 years of EMS experience [165 (43.5%), 296 (78.1%), 240 (63.3%), 258 (68.1%), 182 (48.0%), 203 (53.6%), and 213 (56.2%)], respectively], as shown in Table 1.

### 3.1. Responses Indicating the Level of Motivation among Participants

This study used 19 questions adapted from the GMS survey to investigate what motivates paramedic staff to serve in rural and remote communities. To facilitate the interpretation and comparisons of the paramedic’s responses, we employ a five-point Likert scale with a range of (1) to (5) and an interval that reflected the level of motivation (levels 1 to 2.59, 2.60 to 3.39, and 3.40 to 5 were low, moderate, and high, respectively) [19].

The results indicated a moderate level of motivation with a mean of 3.37 (SD = 0.82) among participants when considering all aspects measured by the questionnaire. The participants generally exhibited high levels of intrinsic motivation with a mean of 3.67 (SD = 0.96), indicating that they were driven by factors such as interest, pleasure, and satisfaction, suggesting a genuine interest in serving in rural and remote communities. Extrinsic motivation, particularly integration and identification (with means of 3.48 (SD = 1.12) and 3.41 (SD = 1.09), respectively), also seemed to be relatively high, suggesting that participants perceive alignment between their personal values and goals and the nature of serving in rural and remote areas. However, there were indications of moderate levels of introjection and external regulation motivation (with means of 3.00 (SD = 1.27) and 3.37 (SD = 1.08), respectively), suggesting some feelings of guilt or external pressure influencing motivation, as shown in Table 2.

Moreover, amotivation scores with a mean of 3.32 (SD = 0.95) suggested that while participants generally see value in serving in rural and remote communities, there are still some who may question its worth or lack a clear reason for doing so. Additionally, the moderate recommendation score, with a mean of 3.37 (SD = 0.82), implied that the participants may not strongly advocate for others to serve in rural and remote communities, possibly reflecting their own mixed feelings about it, as shown in Table 2.

### 3.2. Association between Motivation to Serve in Rural and Remote Communities among Participants and Their Demographic Data

This study employed a Chi-square test to investigate the correlation between demographic variables and motivation to serve in rural and remote communities. The findings indicated significant associations of age (χ^2^ = 167.175, df = 134, *p* = 0.027) and gender (χ^2^ = 98.159, df = 67, *p* = 0.008) with motivation, highlighting that individual under 24 years old and males exhibited higher motivation levels. Conversely, no significant associations were found between job title, employment site, or years of EMS experience and motivation. Overall, while age and gender emerged as significant factors influencing motivation for rural and remote work, other demographic factors such as marital status, education, job title, employment site, and years of EMS experience did not display a notable correlation with motivation in this context, as shown in Table 1.

Furthermore, Cramer’s V coefficients revealed moderate associations of age (V = 0.470) and gender (V = 0.509) with motivation for rural service. Younger individuals and males showed a stronger inclination towards rural service. While other demographic factors also displayed associations, only age and gender reached statistical significance. Further investigation is needed to understand additional factors shaping this relationship.

### 3.3. Combined Analysis of ANOVA Test Results and Multiple Comparisons for Motivation to Serve in Rural and Remote Communities

ANOVA was used to compare mean scores across different demographic groups (age, gender, marital status, education, job title, employment site, and years of EMS experience) to identify if there were significant differences in motivation scores based on these factors. We conducted Levene’s test prior to the ANOVA to assess the assumption of homogeneity of variance. The findings of this study indicated that age, gender, marital status, and years of EMS experience are significant factors influencing motivation for rural and remote work [(F = 6.429, *p* = 0.002), (F = 11.526, *p* = 0.001), (F = 9.311, *p* = 0.002), and (F = 7.635, *p* = 0.001) respectively], while the education level, job title, and employment site do not significantly influence motivation in this context, as shown in Table 3.

Moreover, we conducted Student’s *t*-test for independent samples to explore the association between motivation scores and demographic variables like gender and marital status. The findings revealed significant associations between motivation scores and both gender (t = 3.395, df = 377, *p* = 0.001) and marital status (t = 3.051, df = 377, *p* = 0.002) at the *p* < 0.05 level. As such, unmarried individuals and males exhibited higher motivation levels.

In addition, Scheffe’s post hoc test was used to reveal which specific groups significantly differed in motivation levels. The findings suggested that there were significant differences in motivation to serve in rural and remote communities across different age groups. Specifically, individuals aged 24–30 years exhibited markedly lower motivation compared to both their younger and older counterparts (F = 6.429, *p* = 0.002 *). Furthermore, individuals with less than 5 years of EMS experience displayed diminished motivation relative to their peers with 5–10 years of experience (F = 7.635, *p* = 0.001 *), as shown in Table 3. These results underscore the importance of targeted interventions and support mechanisms, particularly for younger individuals and those in the early stages of their EMS careers, to reinforce motivation and foster retention in rural and remote healthcare settings.

## 4. Discussion

The findings of this study provide valuable insights into the motivations of paramedics to serve in rural and remote communities. With a response rate of 84.2%, this study garnered significant participation, enabling a robust analysis of paramedics’ motivations across various demographic factors. The demographic profile of the participants, predominantly consisting of younger, male, unmarried individuals with bachelor’s degrees and less than 5 years of EMS experience, reflects a common trend in the paramedic profession. Such demographics are often associated with entry-level positions or early career stages within the field.

The overall moderate level of motivation among participants in our study suggests a nuanced blend of intrinsic and extrinsic motivators driving paramedics’ commitment to rural and remote service. High levels of intrinsic motivation signify a genuine interest and satisfaction derived from serving in these communities. Conversely, moderate levels of introjected and external regulation suggest the presence of external pressures or feelings of obligation influencing motivation. Our findings are in line with those of other studies showing that intrinsic motivation has a major influence on healthcare providers’ work engagement. This implies that improving work engagement requires cultivating intrinsic motivation. It also emphasizes how internalizing extrinsic motivation can enhance an employee’s work connection and how employees frequently consider both intrinsic and extrinsic motivations when determining their level of job satisfaction [20,21,22]. In addition, another study revealed that healthcare providers showed moderate external regulation for extrinsic motivation but high levels of intrinsic motivation, identified regulation, and introjected regulation [22]. Moreover, one study found that about half of healthcare providers have average levels of motivation regarding extrinsic regulation [23]. In contrast to our study, extrinsic motivation components appear to have marginally higher mean scores than intrinsic motivation factors, according to other study findings [14]. Further studies showed that the average scores for both intrinsic and extrinsic motivational factors were moderate. Intrinsic subscale scores varied from moderate to high levels, while extrinsic subscale scores ranged from low to high levels. These studies’ conclusions serve as a warning to policymakers that the procedures and policies to enhance worker motivation must be strengthened, particularly among less experienced staff members [24]. These staff members might feel committed to their work out of a sense of duty to fulfill ethical obligations, or they may be motivated by a combination of social factors, duty, and professional responsibilities [25,26,27].

The significant associations of age and gender with motivation in our study underscore the importance of considering individual characteristics in understanding paramedics’ motivations. Younger individuals and males exhibited higher motivation levels, indicating potential differences in priorities, values, or career aspirations across demographic groups. Furthermore, the influence of years of EMS experience on motivation highlights the role of professional growth and familiarity with the demands of rural and remote service in shaping paramedics’ commitment. Individuals with more experience generally displayed higher motivation levels, suggesting that exposure to diverse situations and challenges enhances the dedication to serving in such communities. Our findings are consistent with those of other studies, which revealed that gender differences impact the relationship between extrinsic and intrinsic motivation. In both male and female groups, extrinsic motivation significantly influences intrinsic motivation, but this influence is slightly stronger in men. Moreover, intrinsic motivation significantly affects job satisfaction in men but not in women [28]. In addition, another study revealed that there was a stronger positive correlation between professional opportunities and motivation among younger employees compared to their older counterparts. This indicates that when workers are presented with greater career prospects, their motivation levels rise [29]. Contrary to our study findings, in a previous investigation, female healthcare providers exhibited significantly higher aggregate ratings for both intrinsic and extrinsic motivational elements compared to their male counterparts. Additionally, female healthcare providers were substantially more satisfied with their work environment, possibilities for advancement, and aspects of their line of work [24]. In addition, another study revealed that as healthcare professionals gain experience, they become more familiar with their work’s challenges and rewards, which enhances their skills and job performance, positively impacting motivation levels [30]. The mixed results might be explained by the fact that studies contain different samples and are subject to situational and sociocultural factors [31,32].

Interestingly, factors such as the job title, employment site, and education level did not significantly impact motivation. This suggests that intrinsic drivers, personal values, and individual experiences play a more significant role in shaping motivation than external factors related to job roles or educational attainment. The results of our study are in line with those of other investigations that did not discover any relationships between the motivation levels of healthcare professionals and sociodemographic factors such age, income, marital status, credentials, human resources, kind of employment, and hours worked per week. Professionals’ age appeared to have a negative link with their level of motivation, but this relationship was not statistically significant [33]. Contrary to our findings, other studies revealed relationships among variables across various sample characteristics like gender, age, job position, and employment status. Extrinsic motivation predicts higher intrinsic motivation and job satisfaction, with intrinsic motivation mediating this relationship, along with factors such as gender, age, job position, and employment status [28]. This could be thought to be related to workers’ work–life experiences and how cultural norms, heightened expectations in particular institutional settings, and government support for the work–life balance affect the nature of the work interface [34].

Furthermore, Scheffe’s post hoc analysis revealed that adults in their mid-twenties exhibited markedly lower motivation compared to both their younger and older counterparts, indicating a potential age-related trend where individuals approaching mid-adulthood may have different priorities, perspectives, or circumstances that influence their willingness or motivation to serve in rural and remote areas. Furthermore, the influence of the years of EMS experience on motivation highlights the role of professional growth and familiarity with the demands of rural and remote service in shaping paramedics’ commitment. Individuals with less EMS experience displayed diminished motivation relative to their peers with more years of experience, suggesting that exposure to the challenges and experiences of working in EMS over time can increase paramedics’ dedication to serving in these areas. Our study’s findings are consistent with previous research that showed how the practice environment and motivation level were positively impacted by having more work experience, feeling more autonomous, collaborating with other healthcare providers, and being satisfied with the pay and opportunities for professional growth [35]. Interestingly, adults in their mid-twenties exhibited lower motivation compared to their younger and older counterparts, suggesting that age influences proactive work behavior through intrinsic motivation [36,37]. However, contrary to the findings of our study, there is still a base level of worker productivity even when motivation and work experience are taken into account. This suggests that productivity is influenced by other inherent characteristics as well, setting a baseline level of performance independent of these particular effects [38]. This might be explained by the fact that different life stages may bring varying priorities and responsibilities that impact motivation levels [39].

Overall, our study findings highlight the multifaceted nature of paramedics’ motivations to serve in rural and remote communities, influenced by a combination of intrinsic passion, external pressures, and individual characteristics. Understanding these dynamics is crucial for designing targeted interventions to enhance recruitment, retention, and support for paramedics in these challenging environments.

### Limitations and Practical Implications

While this study provides valuable insights into the motivations of paramedics to serve in rural and remote communities, several limitations should be acknowledged. This study’s sample predominantly comprises younger, male, and less experienced paramedics, potentially limiting the generalizability of the findings to more diverse demographic groups within the profession. Furthermore, this study’s cross-sectional design provides a snapshot of paramedics’ motivations at a single point in time, precluding causal inferences or assessments of how motivations may evolve over time or in response to changing circumstances. Moreover, this study’s findings may be specific to the geographic region or healthcare system under study, limiting their applicability to paramedics serving in different contexts or jurisdictions. Addressing these limitations through larger, more diverse samples, longitudinal designs, and incorporating qualitative methods could strengthen future research on paramedics’ motivations in rural and remote communities, providing a more comprehensive understanding of this complex phenomenon.

The implications of this research study suggest several key points for healthcare organizations and policymakers. Firstly, recognizing the motivations of paramedic staff in rural and remote communities is crucial for addressing the persistent shortage of healthcare workers in these areas. Understanding the balance between intrinsic and extrinsic motivations, particularly among younger, male paramedics, can inform recruitment and retention strategies. Additionally, the significant predictive factors identified, such as age, gender, and EMS experience, highlight the importance of tailored support and flexible arrangements to enhance motivation and job satisfaction. By acknowledging individual traits and providing targeted interventions, healthcare organizations can better meet the needs of paramedic staff serving in rural and remote communities, ultimately improving the quality of healthcare delivery in these areas.

## 5. Conclusions

This study sheds light on paramedics’ motivations in rural and remote areas, with younger, male individuals displaying higher motivation levels. While intrinsic motivation was high, external pressures also influenced motivation. Professional growth and familiarity with the job demands enhanced motivation, indicating that experience plays a crucial role in commitment. Our study underscored the importance of age and EMS experience in shaping motivation levels. As such, understanding individual characteristics is crucial for designing targeted strategies and interventions to enhance motivation among healthcare professionals in rural and remote settings.

To enhance paramedics’ motivation, we recommend fostering intrinsic motivation through continuous professional growth opportunities and tailored support programs considering age and gender. For instance, implementing recognition systems and flexible work arrangements can create a supportive environment. Moreover, prioritizing career development and alleviating external pressures will improve retention and care quality in rural areas. Nonetheless, further qualitative investigation is needed to understand these motivations better and inform staffing and retention strategies.

## Figures and Tables

**Table 1:** The demographic characteristics of participants, the Chi-square test results for motivation to serve in rural and remote communities, and participants’ demographic data (n = 379).

Characteristic	n (%)	χ^2^	Cramer’s V	df	*p*-Value
Age					
Less than 24 years old	165 (43.5)	167.175	0.470	134	0.027 *
24–30 years old	102 (26.9)				
More than 30 years old	112 (29.6)				
Gender					
Male	296 (78.1)	98.159	0.509	67	0.008 *
Female	83 (21.9)				
Marital status					
Not married	240 (63.3)	69.096	0.427	67	0.406
Married	139 (36.7)				
Education					
Less than diploma	15 (4.0)	192.891	0.412	201	0.647
Diploma degree	85 (22.4)				
Bachelor’s degree	258 (68.1)				
Post-graduate degree	21 (5.5)				
Job title					
Health assistant	16 (4.2)	213.187	0.433	201	0.265
EMS—Technician	84 (22.2)				
EMS—Paramedic	182 (48.0)				
EMS student	97 (25.6)				
Employment site					
Urban	203 (53.6)	147.955	0.442	134	0.194
Rural	67 (17.7)				
Unemployed	109 (28.8)				
Years of EMS experience					
Less than 5 years	213 (56.2)	136.819	0.425	134	0.416
5–10 years	96 (25.3)				
More than 10 years	70 (18.5)				

* Significant at *p* < 0.05.

**Table 2:** Mean scores of participants’ responses to the Global Motivation Scale (GMS) questionnaire.

#	Statement	Mean ± SD	Motivation Level
Intrinsic motivation			
1.	Because I like making interesting discoveries.	3.65 ± 1.14	High
2.	For the pleasure of acquiring new knowledge.	3.82 ± 1.15	High
3.	For the pleasant sensations I may feel while I am serving in rural and remote communities.	3.54 ± 1.22	High
**Overall Intrinsic Motivation subscale**		**3.67 ** **± 0.96**	**High**
Extrinsic motivation			
	Integration		
1.	Because by serving in rural and remote communities, I may live in line with my deepest principles.	3.51 ± 1.24	High
2.	Because by serving in rural and remote communities I may fully expressing my deepest values.	3.42 ± 1.30	High
3.	Serving in rural and remote communities may reflect what I value the most in life	3.51 ± 1.23	High
**Overall Integration subscale**		**3.48 ** **± 1.12**	**High**
	Identification		
1.	Serving in rural and remote communities may help me become the person I aim to be.	3.47 ± 1.21	High
2.	Because I may choose rural and remote communities as means to attain my objectives.	3.41 ± 1.26	High
3.	Because I may choose rural and remote communities in order to attain what I desire.	3.33 ± 1.29	Moderate
**Overall Identification subscale**		**3.41 ** **± 1.09**	**High**
	Introjection		
1.	Because otherwise I would feel guilty for not serving in rural and remote communities.	3.04 ± 1.36	Moderate
2.	Because I would beat myself up for not serving in rural and remote communities.	3.00 ± 1.38	Moderate
3.	Because I would feel bad if I do not serve in rural and remote communities.	2.94 ± 1.40	Moderate
**Overall Introjection subscale**		**3.00 ** **± 1.27**	**Moderate**
	External regulation		
1.	Because I want to be viewed more positively by certain people.	3.20 ± 1.29	Moderate
2.	In order to show others what I am capable of.	3.47 ± 1.29	High
3.	In order to attain prestige.	3.42 ± 1.28	High
**Overall External Regulation subscale**		**3.37 ** **± 1.08**	**Moderate**
**Overall Extrinsic Motivation subscale**		**3.31 ** **± 0.95**	**Moderate**
Amotivation			
1.	It does not make a difference whether I serve in rural and remote communities or urban.	3.39 ± 1.33	Moderate
2.	I do not have a good reason for serving in rural and remote communities.	3.49 ± 1.24	High
3.	I believe serving in rural and remote communities is not worth the trouble.	3.09 ± 1.30	Moderate
**Overall Amotivation subscale**		**3.32 ** **± 0.95**	**Moderate**
Recommendation			
1.	I recommend other colleagues serve in rural and remote communities.	3.38 ± 1.16	Moderate
**Overall Global Motivation Scale (GMS) scores**		**3.37 ** **± 0.82**	**Moderate**

The bold values provided in this table are the overall mean and SD (standard deviation) for each Global Motivation Scale (GMS) subscale.

**Table 3:** Combined analysis of ANOVA test results and multiple comparisons for motivation to serve in rural and remote communities.

Characteristic	Mean ± SD	F	*p*-Value	Mean Difference	Sig.
Age					
Less than 24 years old	3.35 ± 0.761	6.429	0.002 *	−0.233 ^a^	0.053
24–30 years old	3.19 ± 0.750			−0.396 ^b^	0.001 *
More than 30 years old	3.58 ± 0.943			0.396 ^c^	
Gender					
Male	3.30 ± 0.815	11.526	0.001 *		
Female	3.64 ± 0.823				
Marital status					
Not married	3.28 ± 0.803	9.311	0.002 *		
Married	3.54 ± 0.846				
Education					
Less than diploma	3.15 ± 0.830	1.137	0.334		
Diploma degree	3.50 ± 0.850				
Bachelor’s degree	3.35 ± 0.825				
Post-graduate degree	3.32 ± 0.760				
Job title					
Health assistant	3.36 ± 1.108	0.681	0.564		
EMS—Technician	3.47 ± 0.851				
EMS—Paramedic	3.38 ± 0.765				
EMS student	3.29 ± 0.874				
Employment site					
Urban	3.37 ± 0.849	0.202	0.817		
Rural	3.43 ± 0.708				
Unemployed	3.35 ± 0.862				
Years of EMS experience					
Less than 5 years	3.25 ± 0.790	7.635	0.001 *	−0.389 ^d^	0.000 *
5–10 years	3.63 ± 0.788			0.389 ^e^	
More than 10 years	3.41 ± 0.915				

* Significant at *p* < 0.05. ^a^ Significant mean difference between less than 24 years old and more than 30 years old. ^b^ Significant mean difference between 24–30 years old and more than 30 years old. ^c^ Significant mean difference between 24–30 years old and more than 30 years old. ^d^ Significant mean difference between less than 5 years and 5–10 years. ^e^ Significant mean difference between less than 5 years and 5–10 years.

## Data Availability

All data relevant to this study are included in the article.

## Supplementary Materials

The following supporting information can be downloaded at https://www.mdpi.com/article/10.3390/healthcare12111062/s1, Table S1: Demographic data; Table S2: The Global Motivation Scale (GMS) questionnaire.
