# Supplementary material for: What Drives Paramedics to Serve in Rural and Remote Communities?
Source: Healthcare (Basel). 2024 May 23;12(11):1062. doi: 10.3390/healthcare12111062 (PMC11172002; doi:10.3390/healthcare12111062)
Supplement: Supplementary file 1 [file healthcare-12-01062-s001.zip › healthcare-3001440-supplementary.pdf]

Supplementary Materials

Survey Questionnaire

Table S1

Part one: Demographic Data

**Instruction:** Please check your answers	
**Characteristic**	**Answer**
Age	Less than 24 years old<br>24-30 years old<br>More than 30 years old
Gender	Male<br>Female
Marital status	Not married<br>Married
Education	Less than diploma<br>Diploma degree<br>Bachelor's degree<br>Post-graduate degree
Job title	Health assistant<br>EMS – Technician<br>EMS – Paramedic<br>EMS student
Employment site	Urban<br>Rural<br>Unemployed
Years of EMS experience	Less than 5 years<br>5 – 10 years<br>More than 10 years

Table S2

Part two: The Global Motivation Scale (GMS) Questionnaire

Instruction: Please indicate to what extent each of the following statements corresponds to the reasons why you may be motivated to work in rural and remote communities using a 5-point Likert scale: (1) strongly disagree; (5) strongly agree.

#	**Statement**
**Intrinsic Motivation**	
1.	Because I like making interesting discoveries.
2.	For the pleasure of acquiring new knowledge.
3.	For the pleasant sensations I may feel while I am serving in rural and remote communities.
	**Overall Intrinsic Motivation subscale**
**Extrinsic motivation**	
	**Integration**
1.	Because by serving in rural and remote communities, I may live in line with my deepest principles.

2. Because by serving in rural and remote communities I may fully expressing my deepest values.
3. Serving in rural and remote communities may reflect what I value the most in life

Identification

1. Serving in rural and remote communities may help me become the person I aim to be.
2. Because I may choose rural and remote communities as means to attain my objectives.
3. Because I may choose rural and remote communities in order to attain what I desire.

Introjection

1. Because otherwise I would feel guilty for not serving in rural and remote communities.
2. Because I would beat myself up for not serving in rural and remote communities.
3. Because I would feel bad if I do not serve in rural and remote communities.

External regulation

1. Because I want to be viewed more positively by certain people.
2. In order to show others what I am capable of.
3. In order to attain prestige.

Amotivation

1. It does not make a difference whether I serve in rural and remote communities or urban.
2. I do not have a good reason for serving in rural and remote communities.
3. I believe serving in rural and remote communities is not worth the trouble.

Recommendation

1. I recommend other colleagues serve in rural and remote communities.
